# Long-term psychological effects of a no-sedation protocol in critically ill patients

**DOI:** 10.1186/cc10586

**Published:** 2011-12-13

**Authors:** Thomas Strøm, Mette Stylsvig, Palle Toft

**Affiliations:** 1 Department of Anesthesia and Intensive Care Medicine, Odense University Hospital, Sdr Boulevard 29, 5000 Odense C, University of Southern Denmark, Odense, Denmark

## Abstract

**Introduction:**

A protocol of no sedation has been shown to reduce the time patients receive mechanical ventilation and to reduce intensive care and total hospital length of stay. The long-term psychological effects of this strategy have not yet been described. The purpose of the study was to test whether a strategy of no sedation alters long-term psychological outcome compared with a standard strategy with sedation.

**Methods:**

During intensive care stay, 140 patients requiring mechanical ventilation were randomized to either no sedation or sedation with daily interruption of sedation. This study was done as a single-blinded cohort study. After discharge, patients were interviewed by a neuropsychologist assessing quality of life, depression, anxiety, and posttraumatic stress disorder.

**Results:**

Two years after randomization, 38 patients were eligible for interview, and 26 patients were interviewed (13 from each group). No difference was found with respect to quality of life (Medical Outcome Study, 36-item short-form health survey). Both mental and physical components were nonsignificant. The Beck depression index was low in both groups (one patient in intervention group versus three patients in the control group were depressed, *p *= 0.32). Evaluated with the Impact of Events Scale, both groups had low stress scores (one in the intervention group versus two in the control group had scores greater than 32; *p *= 0.50). State anxiety scores were also low (28 in the control group versus 30 in the intervention group, *p *= 0.58).

**Conclusions:**

Our data suggest that a protocol of no sedation applied to critically ill patients undergoing mechanical ventilation does not increase the risk of long-term psychological sequelae after intensive care compared with standard treatment with sedation.

## Introduction

Critically ill patients admitted to an intensive care department, undergoing mechanical ventilation, are at high risk of neurocognitive sequelae after intensive care stay [1]. The general practice is to sedate critically ill patients undergoing mechanical ventilation [2]. It is commonly believed that sedation alleviates the stress from intensive care, especially mechanical ventilation. When sedation is used, it is done with the best intention, but it has never been proven that the use of sedation ensures a better psychological outcome. On the contrary, Kress and colleagues [3] reported that a daily wake-up trial might reduce the severity of posttraumatic stress disorder (PTSD). This was a surprising finding, especially because none of the patients from the group undergoing a daily interruption of sedatives could remember being awakened. However, a majority of patients remembered having been admitted to the intensive care department. Jones and colleagues [4] reported that patients with recollections of intensive care stay were less prone to develop PTSD than were patients with no recollections at all. Recently other studies have confirmed that less sedation does not increase the severity of long-term psychological outcome [5,6].

We previously published a study showing that a protocol of no sedation reduced the time patients received mechanical ventilation and reduced the intensive care and total hospital length of stay [7]. Concerns have been raised that our method with only bolus doses of morphine and no sedation carries a higher risk of psychological trauma than does standard care with sedation [8-10]. A study investigating the psychological effects of a no-sedation strategy has been warranted [8-10]. We conducted an *a priori *planned prospective study interviewing all available patients from our original study to evaluate the psychological long-term effect after intensive care stay. This is the first study reporting the psychological effects of a no-sedation strategy for critically ill patients undergoing mechanical ventilation. Our main hypothesis was that a strategy with no sedation does not worsen long-term psychological and functional outcomes compared with a standard strategy of sedation and daily interruption of sedatives. Some of the results of this study have been previously reported in the form of an abstract.

## Materials and methods

### Settings and patients

In this *a priori *planned prospective part of a randomized controlled trial, we assessed long-term psychological effects after hospital discharge in patients enrolled in our no-sedation trial. Patients were recruited from both medical and surgical sections of the 18-bed multidisciplinary, closed Intensive Care Department at Odense University Hospital, Odense, Denmark. All parts of the study were approved by the local Scientific Ethics Committee. Written informed consent was obtained from every patient or the patient's representatives. The study was registered in ClinicalTrials.gov, number NCT00466492. Adult patients in need of mechanical ventilation for more than 24 hours were enrolled in the study. Exclusion criteria were younger than 18 years, pregnancy, increased intracranial pressure, or needed sedation (for example, seizures or therapeutic hypothermia).

### Intervention

The 140 patients were randomized to two groups: the intervention group receiving no sedation but bolus doses of morphine, or the control group receiving continuous sedation and a daily interruption of sedatives. Patients who were disconnected from mechanical ventilation within 48 hours (successfully weaned or died) were not included in the statistical analysis. Besides bolus doses of morphine, as deemed necessary by the nurse, patients in the awake intervention group did not receive sedation during their intensive care stay. If delirium was clinically suspected, haloperidol was used. The control group was sedated with propofol for 48 hours and then changed to midazolam. Patients in the control group were sedated to a Ramsay score of 3 to 4, and on a daily basis, interruption of sedatives was performed as described by Kress and colleagues [11]. Besides sedation, the control group was also given morphine as bolus doses. The department's nurse-to- patient ratio is 1:1 for all patients. If patients were uncomfortable, it was possible to call for an extra person to reassure and comfort the patients verbally. If a patient from the no-sedation intervention group could not tolerate being awake, sedation could be started for 6 hours and then turned off. If this happened 3 times, patients were treated with sedation, as in the sedated control group. Data were treated according to randomization; no crossover was allowed (intention to treat). If at all possible, patients from both groups were mobilized on a daily basis. After intubation, all patients were changed as quickly as possible from controlled to support ventilation. A full description of the study protocol can be found in the original study [7].

### Psychological outcome

For this psychological part of the study, all patients still alive were contacted by telephone. Patients unable to participate in a short telephone interview (dementia, and so on) were excluded. Patients were offered an interview with a clinical neuropsychologist (M.S.) at the hospital. The neuropsychologist was not aware of the randomized treatment (sedation or no sedation). Besides refund of travel expenses, patients did not receive any financial reimbursement or any treatment.

Before the interview, patients received the Medical Outcome Study 36-item short-form health survey (SF-36) questionnaire and were asked to fill it out [12]. It was collected at the interview. Each patient's previous psychiatric illness before and after randomization and the use of psychotropic medication before and after intensive care were recorded at the interview. Time from randomization to interview and time from discharge from hospital to interview was recorded. At the interview, patients were evaluated by using Beck Depression Inventory 2 score (BDI-II) [13], State Anxiety Inventory [14], Revised Impact of Event Scale [15], and Post-Traumatic Stress Syndrome 10-Questions Inventory (PTSS-10) [16] (see an overview of the tests in Table 1). Besides the psychological tests, a physician (T.S.) asked the patients seven questions about their intensive care stay. The questions were a modification of the ICU memory tool [17]. The questions were (1) whether patients recalled being admitted to intensive care, (2) remembered being awakened, (3) if they received sufficient rest, (4) whether they had nightmares, (5) if they had pain, (6) whether they had trouble breathing, and (7) whether they were still affected by their intensive care stay.

**Table 1:** Psychological assessments

Test	Description	Area measured	Scoring
Medical Outcome Study 36 item short-form health survey (SF-36)[12]	8-scale profile of functional health and well-being scores as well as psychometrically based physical and mental health summary measures	Generic quality of life	36 questions across eight domains; range, 0-100, with low scores indicating poor quality of life
Beck Depression Inventory 2 (BDI-II)[13]	Screening tool to assess severity of depression	Depression	21 questions ranging from 0 to 3 (total range, 0-63). A score > 10 is suggestive of depression
State Anxiety Inventory [14]	Assess current anxiety	Anxiety	20 questions ranging from 1 (not at all) to 4 (very much so) (total range, 20-80).
Revised Impact of Event Scale (IES-R) [15]	Assess current subjective distress for any specific life event	PTSD symptoms	22 questions ranging from 0 (not at all) to 4 (extremely) (total range, 0-88). A score > 32 is suggestive of PTSD
Post-Traumatic Stress Syndrome 10-Questions Inventory (PTSS-10) [16]	Screening tool to assess the presence of PTSD symptoms	PTSD symptoms	10 questions ranging from 0 to 7 (total range, 0-70). A score > 35 is suggestive of PTSD

### Statistical analysis

No separate power analysis was done for this part of the study. Continuous data are presented as median values with interquartile range. Categoric data are presented as numbers and percentages. Continuous data were analyzed by using the Wilcoxon rank-sum test. Categoric data were analyzed by using the χ^2 ^or the Fisher Exact test, as appropriate. The SF-36 questionnaire is an eight-scale profile of functional health and well-being scores, as well as psychometrically based physical and mental health summary measures. A *p *value less than 0.05 was considered significant. All tests were performed by using: StataCorp 2009; Stata Statistical Software: Release 11, College Station, TX: StataCorp LP.

## Results

**Figure 1:**
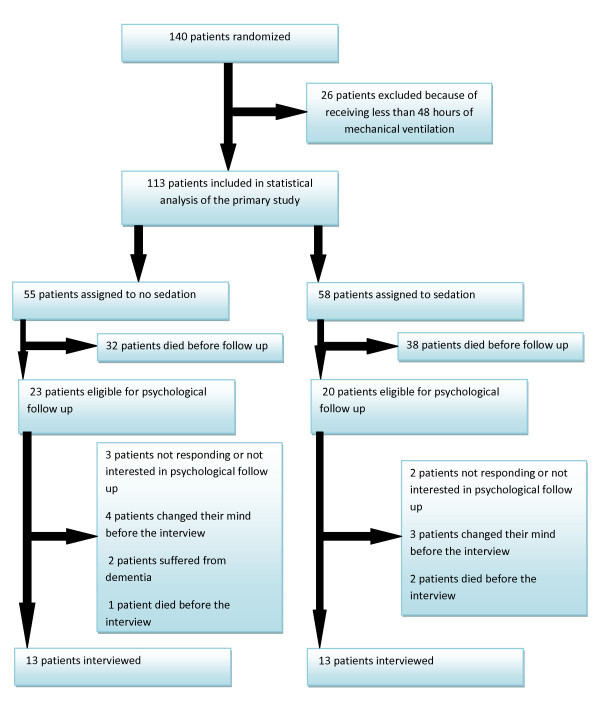
**Consort diagram showing the flow of patients in the trial**.

Of the randomized 140 patients, 70 patients died before psychological follow-up. No statistically significant difference in mortality in the follow-up period was found between the two groups. Forty-three patients were eligible for psychological follow-up. Five patients did not respond or were not interested in psychological follow-up. Thirty-eight patients initially agreed to participate in a psychological follow-up. Twelve patients changed their minds, had dementia, or died before the interview. In total, 26 patients were interviewed. A Consort diagram of the patient flow is shown in Figure 1. All patients but one were interviewed at the hospital. One patient was interviewed in his home. Demographic data for these patients are presented in Table 2. No statistically significant difference was found with respect to age, gender, weight, APACHE II, SAPS II, or SOFA (day 1) between these patients. In both groups, more female than male patients were interviewed. The number of ventilator-free days in a 28-day period was again higher in the nonsedated group compared with the sedated control group. It did not, however, reach statistical significance in this small cohort of patients. The cumulative morphine use during the time of mechanical ventilation was higher in the nonsedated group but neither reached a statically significant difference. Both propofol and midazolam use was again higher in the sedated control group compared with the nonsedated intervention group (*p *= 0.0127 and *p *= 0.0029; Table 2). The use of haloperidol was as previously higher in the nonsedated group compared with the sedated intervention group (*p *= 0.0125), but still the cumulative dose was very low. Times from randomization to interview and hospital discharge to interview were without any difference between the two groups (almost 2 years (Table 2)).

**Table 2:** Baseline data

	No Sedation (*n *= 13)	Sedation (*n *= 13)	*p *value
Age (years)	71 (58-74)	63 (56-67)	0.33
Female	9 (69%)	8 (62%)	0.5
Weight (kg)	80 (75-90)	96 (70-103)	0.26
Apache II	20 (16-29)	25 (21-26)	0.20
SAPS II	41 (34-49)	46 (32-49)	0.63
SOFA (at day 1)	7 (5-9)	7 (5-10.5)	0.91
Ventilator-free days (28 days)	23.15 (19.00-25.35)	16.13 (3.92-22.67)	0.12
Morphine (mg/kg/h)^a^	0.0088 (0.0039-0.01676)	0.0047 (0.0030-0.0060)	0.24
Propofol (mg/kg/h)^ab^	0 (0-1.2553)	1.3996 (0.5178-2.0408)	0.0127
Midazolam (mg/kg/h)^a^	0 (0-0)	0.0135 (0-0.0405)	0.0029
Haloperidol (mg/kg/h^a ^	0.0039 (0-0.0202)	0 (0-0)	0.0125
Time from randomization to psychological interview (years)	1.78 (1.46-2.10)	2.04 (1.55-2.29)	0.32
Time from hospital discharge to interview (years)	1.72 (1.42-2.05)	1.92 (1.47-2.20)	0.49

Data are expressed in number (%) or median (IQR). APACHE II, acute physiology and chronic health evaluation; SAPS II, simplified acute physiology score; SOFA, sequential organ-failure assessment. ^a^While receiving mechanical ventilation. ^b^Dose during a maximum of 48 hours of treatment.

Patients who reported having psychological problems before intensive care stay also reported having this at follow up. One patient from the awake intervention group compared with three patients in the sedated control group reported that they had been without psychological problems before ICU admission but did have psychological problems after hospital discharge. This did not reach statistical significance (Table 3).

**Table 3:** Long-term outcomes

	No sedation (*n *= 13)	Sedation (*n *= 13)	*p *value
Psychological problems before admission to ICU	1 (8%)	3 (23%)	0.59
Psychological problems after hospital discharge	2 (15%)	6 (46%)	0.20
Use of psychological medication before admission to ICU	1 (8%)	3 (23%)	0.593
Use of psychological medication after hospital discharge	2 (15%)	2 (15%)	1.00
SF-36			
Mental component	58 (51-61)	52 (37-60)	0.46
Physical component	39 (31-46)	40 (31-43)	0.85
BDI-II			
Overall score	3 (1-7)	3 (1-11)	0.61
Number of patients being depressed (score > 10)	1 (8%)	4 (31%)	0.32
State Anxiety Inventory	48 (45-50)	50 (45-53)	0.58
Impact of Events Scale			
Overall score	4 (2-8)	2 (0-11)	0.41
Number of patients with PTSD (Score > 32)	1 (8%)	2 (15%)	0.50
PTSS-10			
Nightmares	9 (69%)	6 (46%)	0.23
Anxiety and nightmares	3 (23%)	4 (31%)	1.00
Pain	1 (8%)	0 (0)	1.00
Trouble breathing	2 (15%)	5 (38%)	0.37
Sum of B questions	3 (0-6.5)	10 (6-17)	0.09
Number of patients suggestive of PTSD (Score > 35)	1 (8%)	0 (0)	0.14
Modified ICU memory tool			
Remember ICU (yes)	9 (69%)	8 (62%)	0.68
Remember Wake-up (yes)	2 (15%)	5 (38%)	0.37
Sufficient rest (yes)	12 (92%)	12 (92%)	1.00
Nightmares (yes)	8 (62%)	8 (62%)	1.00
Pain (yes)	2 (15%)	1 (8%)	0.50
Trouble breathing (yes)	1 (8%)	0 (0)	1.00
Still affected by ICU (yes)	3 (23%)	1 (8%)	0.29

Data are expressed in median (IQR) or number (%).

Quality of life examined with the SF-36 did not yield any statistical difference between the two groups (Table 3). Compared with age-matched Danish reference values, our patients did have lower scores: mental component Danish reference mean, 55.61 (SD, 8.73) versus overall mean score (26 patients), 51.71 (SD, 13.14), *p *= 0.16 and physical component Danish reference mean, 45.14 (SD, 10.76) versus overall mean score (26 patients), 38.94 (SD, 10.33), *p *= 0.0088 [12].

Although the overall Beck depression score (BDI-II) was low (median value, three in each group), one patient in the awake group versus patients in the sedated group, had a depression score above 10 and thereby suggestive of depression, *p *= 0.32 (Table 3).

With respect to anxiety, no difference between the groups was found evaluated by the State Anxiety Inventory scale test (Table 3).

PTSD was a very rare finding. Revised Impact of Event Scale yielded very low values; only one patient from the intervention group and two patients from the control group had scores above 32, suggestive of PTSD. These low numbers yielded no statistical difference between the groups. The screening tool, Post-Traumatic Stress Syndrome 10-Questions Inventory, suggested a very low occurrence of PTSD. The sum of B questions was low in both groups: 3 (0 to 6.5) in the nonsedated intervention group versus 10 (6 to 17) in the sedated control group (*p *= 0.09). Overall, only one patient from the intervention group had a score above 35, and this could be suggestive of having PTSD, according to the Post-Traumatic Stress Syndrome 10-Questions Inventory test. For a summary of the results, see Table 3.

Most of the patients from both groups remembered being admitted to the intensive care department (69% from the intervention group versus 62% from the control group, *p *= 0.68). However, more patients from the control group (38%) remembered having been awakened in the intensive care department compared with the nonsedated intervention group (15%), although the results are not statistically significant (*p *= 0.38). The control group was, according to the purpose of the study, not sedated at all. In both groups of patients, two of three recalled having had nightmares during their intensive care stay, but no difference between the two groups (62% in both groups; *p *= 1.00). In both groups, a very low number of patients remembered experiencing pain, trouble breathing, or were still affected by their intensive care stay (Table 2).

## Discussion

With this randomized prospective study, we showed that a strategy with no sedation for critically ill patients undergoing mechanical ventilation appears not to worsen long-term psychological and functional outcomes compared with a standard strategy of sedation and daily interruption of sedatives. Although the number of interviewed patients is low, it is interesting to note that the PTSS-10 scores tend to be lower in the awake intervention group compared with the sedated control group.

Over the last decade, evidence of the beneficial effect of a lower use of sedation has increased. Brook and colleagues [18] reported that a nurse-implemented sedation protocol, including only bolus doses of sedation, could reduce the time patients received mechanical ventilation. Kress and colleagues [11] reported that a daily interruption of sedatives could reduce the time patients received mechanical ventilation compared with continuous sedation. Girard and colleagues [19] reported a beneficial effect of combining interruption of sedatives and a spontaneous breathing trial. Both the Kress and Girard study were followed by long-term psychological evaluations, both showing that less sedation did not worsen the long-term psychological outcome [3,6]. Kress and colleagues actually reported that patients undergoing a daily interruption of sedatives had less posttraumatic stress at follow-up. The psychological follow-up of patients from the Girard study reported a beneficial effect from a combined interruption of sedatives and spontaneous breathing trial at 3 months' follow-up. However, this effect could not be reproduced at 12 months of follow-up. Treggiari and colleagues [5] supported the finding that less sedation reduced the time patients received mechanical ventilation without a worse psychological outcome. Our study showing a beneficial effect of a strategy with no sedation can be seen as an extension of these studies. It implies that further reduction in routine use of sedation has a beneficial effect on the reduction in time receiving mechanical ventilation, reduction in ICU length of stay, and reduction in total hospital length of stay. However, it is important to focus not only on reducing time of mechanical ventilation, intensive care, and hospital length of stay. The psychological outcome also is very important. If a treatment holds a risk of increased long-term psychological sequelae, it would not be acceptable.

Another strength with the present study is that all patients were interviewed by the same experienced neuropsychologist. Other studies mailed the questionnaires to patients without the possibility for patients to ask questions while filling out the question form [5]. To have a neuropsychologist interviewing the patients ensures that no patients are under- or overdiagnosed.

Some limitations deserve mentioning. The study was conducted as a single-center study, which holds a risk that data from this study do not apply to other centers. The prolonged interval of 2 years from hospital discharge to interview might have introduced recall bias. More female than male patients participated in the study. It is not known whether this had any influence on the results. The study by Kress et al also included more female than male patients [3]. The design of the study holds a risk of selection bias; the interviewed patients had a lower APACHE II score (median, 22.5) compared with the entire study group of patients from the original study (median APACHE II score, 26). These patients could also be the least psychologically affected. The fact that we did not find a statistically significant difference with respect to ventilator-free days in this subgroup of patients from the original trial implies that this is a small group of patients. However, Kress and colleagues [3] did not find a significant difference with respect to mechanical ventilation in their follow-up study. We interviewed as many patients as possible, but this of course includes only patients still alive, able, and willing to participate at the time of follow-up. In our study, we interviewed 19% of the original randomized patients (26 of 140 patients). The study by Kress and colleagues involved 21% of patients who had been through a similar treatment but not from the original study (32 of 150 patients). The study by Girard [19] at 12 months involved 18% of the patients (63 of 336 patients). Our fraction of interviewed patients is comparable with earlier studies.

Only one patient from the intervention group had PTSD. This is a lower prevalence of PTSD than reported in the literature, which reports up to 22% to 64% [20,21]. Diagnosing PTSD by clinical interview seems to reduce the observed frequency of PTSD compared with self-report measures [20,21]. All patients in our study were assessed daily during the original trial, regardless of the randomized treatment (sedation or no-sedation), which potentially could decrease the risk of PTSD.

A tendency was found toward lower PTSD scores in the nonsedated intervention group. Because of the small number of patients, a risk of a type 2 error exists, implying that we may have overlooked an actual difference in respect to psychological outcome. A greater number of patients could perhaps have minimized this risk.

In the original study, beside the 1:1 nurse/patient ratio, an extra person was called on at 14 occasions. In the subgroup of patients interviewed, two patients from the sedated control had an extra person at their bedside to comfort them verbally. The extra person was present for 2 days in both cases. Whether this has influenced long-term psychological outcome, perhaps reduced the risk of long-term sequelae, is not known, given the low number of patients.

We have not included patients successfully weaned from mechanical ventilation within 48 hours. These patients were excluded from the statistical analysis in our original study. This was done because we believe that less than 48 hours of mechanical ventilation is too short a time with the randomized treatment to give a clear impression of the effects. This also applies to the psychological follow-up.

We interviewed the patients only once. We therefore have no data on fluctuation over time of the patients' psychological status after intensive care. However, interviewing the patients too soon after hospital discharge might have influenced the results, classifying more patients as having psychological sequelae because they might not yet have fully recovered. Also it is possible that interviewing patients earlier would have shown a beneficial effect from a strategy with no sedation on psychological outcome, like the follow-up study by Girard and colleagues [19]. Interview at an earlier time would have included some patients not alive at the actual later follow-up. Perhaps the increase in sample size would have yielded a statistically significant beneficial effect of a strategy with no sedation on psychological outcome.

## Conclusions

A strategy of no sedation compared with a standard strategy of sedation and a daily interruption of sedatives in critically ill patients undergoing mechanical ventilation resulted in no difference with respect to long-term psychological outcome. Much concern has arisen about the long-term psychological effects of a no-sedation strategy; our data do not support the hypothesis that omission of routine use of sedation to critically ill patients results in a worse psychological long-term outcome.

## Key messages

• A strategy of no sedation for critically ill patients receiving mechanical ventilation seems promising with respect to days on mechanical ventilation, days in the ICU, and days in hospital. However, long-term psychological outcome has not yet been described.

• Two thirds of the patients from both groups remembered being admitted to the ICU.

• Our psychological follow-up study implies that a strategy of no sedation appears not to worsen long-term psychological and functional outcomes compared with a standard strategy of sedation and daily interruption of sedatives.

## Abbreviations

APACHE II: Acute Physiology and Chronic Health Evaluation; BDI-II: Beck Depression Inventory 2; IES-R: Revised Impact of Event Scale; PTSD: posttraumatic stress disorder; PTSS-10: Post-Traumatic Stress Syndrome 10-Questions Inventory; SF-36: Medical Outcome Study 36-item short-form health survey; SOFA: Sequential Organ Failure Assessment

## Competing interests

The authors declare that they have no competing interests.

## Authors' contributions

TS and PT conceived and designed the study. MS did all neuropsychological interviews and testing. TS and PT drafted the manuscript. All authors contributed to the review and revisions of the manuscript. All authors read and approved the final version of the manuscript.
